# Rare oculomotor nerve palsy after interventional treatment of anterior communicating artery aneurysm: A case report

**DOI:** 10.1097/MD.0000000000045571

**Published:** 2025-11-07

**Authors:** Yong Cai, Xingming Zhong

**Affiliations:** a First affiliated Hospital of Huzhou University, Huzhou, Zhejiang Province, China; b The First People’s Hospital of Huzhou, Huzhou, Zhejiang Province, China.

**Keywords:** aneurysm, anterior communicating artery aneurysms, nursing, oculomotor nerve palsy

## Abstract

**Rationale:**

The purpose of this case report is to describe the process of oculomotor nerve palsy (ONP) in a patient after interventional treatment of anterior communicating artery aneurysms, so as to remind clinicians to pay attention to the condition of ONP in such patients.

**Patient concerns:**

A 73-year-old female patient was admitted with spontaneous vomiting for 4 days and had a Glasgow coma score score of 12 after admission. Three hours after admission, the Glasgow coma score score decreased to 5, and subsequent lumbar puncture drainage revealed bloody CSF.

**Diagnoses:**

Cerebral angiography confirmed the presence of 2 anterior communicating aneurysms.

**Interventions:**

Stent-assisted coil embolization (stent model: LVIS3.5–17, coil model: APB-1.5-3-3D, APB-1-2-3D, APB-1-1-HX) was performed. A stent was placed in the A2 segment of the anterior cerebral artery, and the aneurysm was completely embolized by stent-assisted release of 3 coils.

**Outcomes:**

The patient developed persistent unilateral ONP after surgery and was not followed up because the family requested discharge.

**Lessons:**

Anterior communicating artery aneurysms are often mistakenly thought not to cause ONP due to their anatomical relationship. Our case confirms that in rare cases, ACOA aneurysm can also cause ONP, which reminds clinicians that they should think more comprehensively when patients present with ONP.

## 1. Introduction

Aneurysms in the brain are mostly abnormal bulges on the wall of the cerebral vessels, which can lead to aneurysm subarachnoid hemorrhage (aSAH) when ruptured. In cerebrovascular accidents, it is second only to cerebral thrombosis and hypertensive cerebral hemorrhage, ranking third, and the mortality associated with cerebral aneurysm rupture accounts for 25% of all deaths from cerebrovascular diseases.^[1]^ Anterior communicating artery (ACoA) is the most common site of intracranial aneurysms, accounting for 30% to 40% of intracranial aneurysms. ACoA aneurysm is also the most common rupture, accounting for 40% of aSAH.^[2,3]^ Oculomotor nerve palsy (ONP) can occur in a variety of situations, including microvascular disease, aneurysm, trauma, painful ophthalmoplegia, stroke, infection, and diabetes.^[4]^ Intracranial aneurysm is the main cause of unilateral oculomotor nerve paralysis, mainly manifested as diplopia, ptosis, ophthalmoplegia and pupillary dysfunction.^[5]^ SAH-related ONP is usually associated with aneurysms at the junction of the internal carotid artery and the posterior communicating artery. Although third nerve paralysis by ACoA aneurysms has been reported, it is still very rare.^[6]^ This case report describes a patient with a pressure ulcer who underwent coil embolization for ruptured anterior communicating artery aneurysms. On the second day after surgery, he developed persistent fever and mydriasis on 1 side, which brought great challenges to nursing work.

## 2. Case presentation

### 2.1. General data

**Figure 1.**
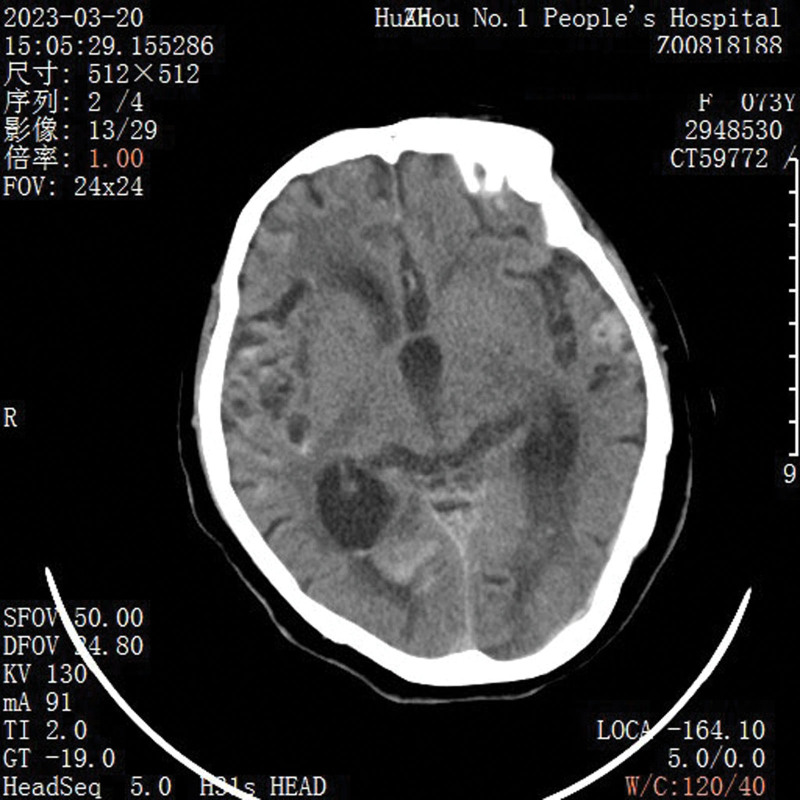
The patient presented with subarachnoid hemorrhage.

A 73-year-old female patient had vomiting for 4 days without obvious inducement. The vomit was gastric content. The patient had progressive disturbance of consciousness for 4 days and was admitted to the emergency department. The patient’s history was brain atrophy and left foot surgery. She was semiconscious when brought to the hospital, and initial physical examination included a heart rate of 100 beats/min, and blood pressure of 170/91 mm Hg. Meanwhile, her neurologic glasgow coma score was 12 and bilateral pupil was 3mm in diameter and sensitive to light reflection. Notably, she was feverish and body temperature was 38.5°C. Emergency computed tomography (CT) (Fig. 1) scan showed signs of subarachnoid hemorrhage. Out-of-Hospital stage 1 left hip pressure sore 5cm * 5cm, right upper limb lateral 1cm * 5cm finger pressure unchanged white erythema.

### 2.2. Course and outcome of diagnosis and treatment

After enrollment, she was given premium care, electrocardiographic monitoring, 5L/min mask oxygen, fasting, and indomethacin bolt 50mg returning anus. Monitor changes in the vital signs. Three hours later, the patient developed coma, Glasgow coma score score decreased to 5 points, limb shaking, blood pressure decreased, body temperature increased, lumbar puncture and drainage of hemorrhagic cerebrospinal fluid. After no improvement in symptomatic treatment, the patient was immediately transferred to EICU for neurological and hemodynamic monitoring. Unfortunately, because of an oversight, the patient’s signs of meningeal irritation were not carefully examined, and her sensory and motor function could not be assessed once she fell into a coma.

**Figure 2.**
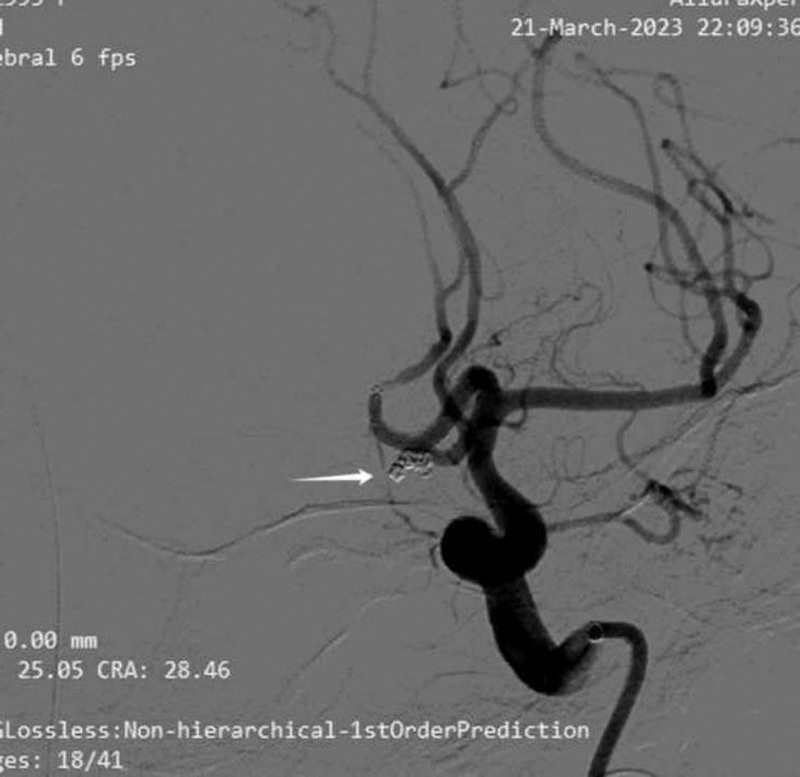
Postoperative embolization of the anterior communicating artery aneurysm.

**Figure 3.**
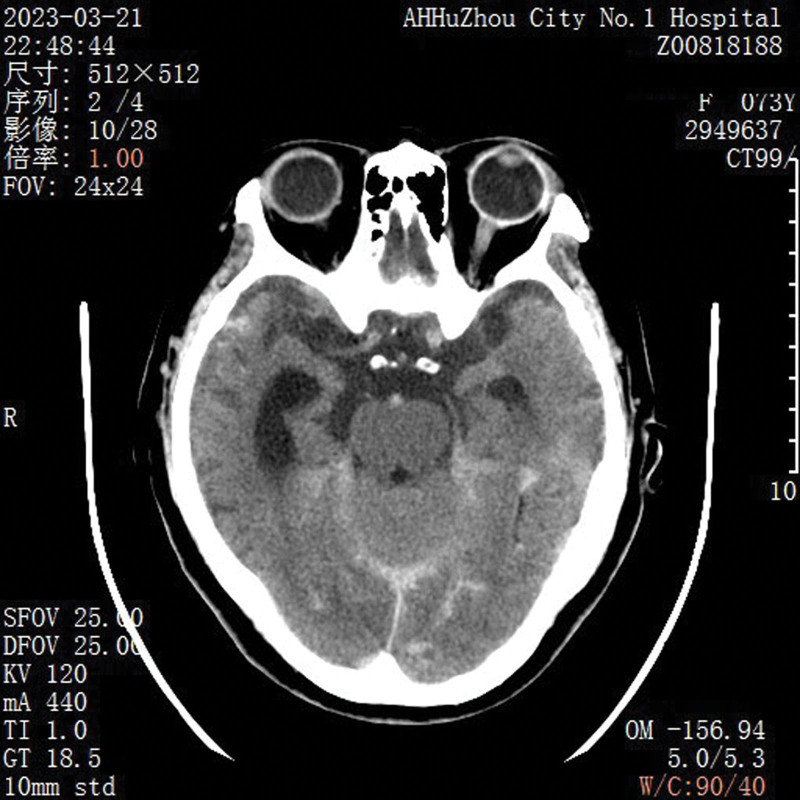
CT images after the procedure were completed and there was no evidence of bleeding. CT = computed tomography.

**Figure 4.**
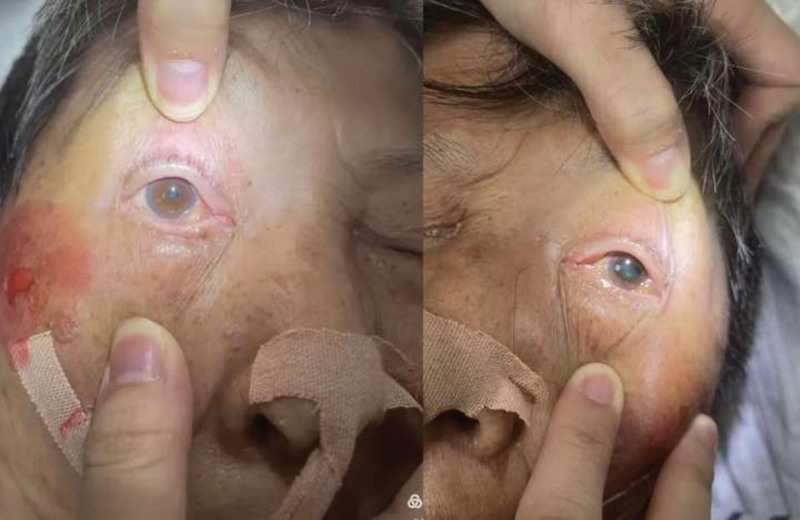
The patient presented with left-sided mydriasis.

**Figure 5.**
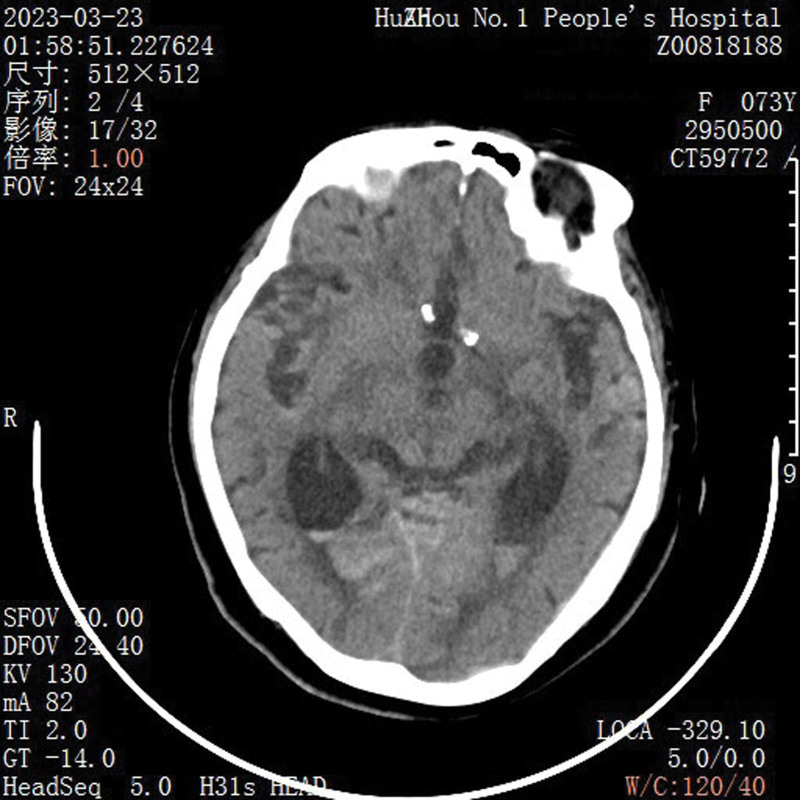
CT taken after the patient’s pupil changed, no evidence of hemorrhage or cerebral herniation. CT = computed tomography.

After multidisciplinary consultation on the next day, cerebral angiography under general anesthesia with tracheal intubation was performed. The results showed that the left common carotid angiography suggested 2 ACoA aneurysms, one with a neck of 1.16 mm, a size of about 1.89 × 1.34 mm, and the other 2.0 × 1.04 mm, and plaque formation at the beginning of the left internal carotid artery with severe stenosis. After multi-angle angiography, combined with the size and shape of the aneurysm, stent-assisted coil embolization was considered. (The stent size was LVIS 3.5–17, and the coil size was APB-1.5-3-3D APB-1-2-3D APB-1-1-HX). The microcatheter of the stent catheter was placed in the A2 segment of the right anterior cerebral artery. After the microcatheter was shaped, the coil was placed in the aneurysm body, and 3 coils were slowly released under the assistance of the stent. The aneurysm was completely embolized, and the A2 segment of the right anterior cerebral artery was partially unclear, which was maintained with tirofiban (6 mL/h). After observation for 5 minutes, the A2 segment of the right anterior cerebral artery was well visualized, and the cerebral angiography was performed again after operation, and the aneurysm cavity was not obviously visualized. The anterior cerebral artery, internal carotid artery, middle cerebral artery and its branches were well visualized. At the end of angiography, the catheter and arterial sheath were withdrawn and sutured with vascular stapler. The puncture point was pressed to stop bleeding and pressure bandage was applied (Fig. 2). Postoperative patients were in a state of sedation, tracheal intubation, and mechanical ventilation. Bilateral pupil 2.0mm, slow light reflex. There was no significant change in head CT (Fig. 3) and no obvious bleeding signs. On the second day after the operation, the patient had left pupil dilation in the early morning, 5.5 mm, and the light reflex disappeared. The right side was 2.0 mm, and the light reflex was slow (Fig. 4). The ear temperature was 37.4 °C to 38 °C, and central venous pressure was 4.5 cm H_2_O. Follow- up CT (Fig. 5) showed no increase in bleeding, and computer tomography angiography examination showed no significant vascular obstruction. Biochemical and cerebrospinal fluid examination showed intracranial infection, and lumbar cistern drainage was performed to control infection and symptomatic treatment.

The patient’s condition progressed rapidly after admission, and the symptoms did not improve significantly after taking appropriate therapeutic measures and nursing measures such as respiratory support, intestinal nutrition, temperature management and skin protection. She had recurrent heat, ear temperature of 37.5°C to 39°C, constant coma, bilateral calf inter-muscle vein thrombosis, occasional facial and lower extremity paroxysm twitch, the body was scattered bruising spots. On the 28th day after surgery, the pupil dilated on the left side of the patient was briefly constricted (2.0mm) and then dilated again. On the 34th day of the operation, the patient’s family requested discharge and failed to follow up.

## 3. Discussion

Intracranial aneurysm is the most common cause of spontaneous subarachnoid hemorrhage. It has an acute onset, rapid changes and can cause cerebral hernia, with high disability and mortality.^[7,8]^ The anterior communicating artery (Acoma), observed in 25% of cases, is the most common location of intracranial aneurysms.^[9]^ Routine treatments for ruptured or unruptured intracranial aneurysms include surgical clipping or intravascular intervention. Surgical clipping means blocking intracranial aneurysms and cerebral circulation blood flow with a specific metal clip to reduce aneurysm pressure on adjacent tissues. Intravascular intervention, such as appropriate embolization of the aneurysm with a coil, can reduce the blood supply of the aneurysm to prevent rupture and bleeding^.[10]^ Compared to surgical clamping, spring coil embolism is less invasive, recovery time faster and has a relatively low mortality rate.^[11]^ However, detailed morphological analysis should be carried out to select the best treatment.

ACoA is located in the cerebral artery ring and has the function of communicating bilateral anterior cerebral arteries. It is an important part of the intracranial artery. Neuro-ophthalmic manifestations that may arise from ACoA aneurysms include compression, ischemia, or bleeding involving the anterior visual pathway. It leads to monocular vision loss, junctional dark spots, or bilateral temporal hemianopia secondary to optic chiasm compression, accompanied by orbital pain and headache.^[12]^ But ACoA aneurysms causing acute third nerve palsy are very rare. In the case of subarachnoid hemorrhage induced by aneurysm rupture, ONP may be caused by cerebral vasospasm, compression of subarachnoid hematoma, direct stimulation of blood to nerves, and nerve ischemia.^[13]^ During hospitalization, the patient received a 30mg intravenous infusion of papaverine needle group to improve cerebral vasospasm, sustained 1mg/h micropump intravenous infusion of nimodipine 10mg to improve cerebral vasospasm, and intravenous infusion of valproate 1.2g at a rate of 50mg/h through the injection pump to treat epilepsy. Studies have shown that the ONP caused by aneurysms, the pupil was initially only typically dilated and fixed, but within 2 to 4 weeks, it usually contracted and almost completely recovered within 3 to 6 months.^[14–16]^ In this case, the patient’s left dilated pupil was briefly reduced to 2.0mm at 4 weeks after surgery and then dilated again. Because the patient has been in a coma and is unable to observe more pupils, it is clinically difficult to determine the exact pathophysiological mechanism that causes this case.

Intracranial infection and lung infection are present in this patient. The lumbar cistern drainage controlled the intracranial infection, but the patients still had recurrent fevers for a long time. The nursing measures such as turning over and knocking back, sputum suction, oral cleaning and the application of antibiotics failed to significantly improve the symptoms of the patients. Blood routine examination showed a rebound of inflammatory markers. After discussion by neurosurgeons, it was considered that there maybe damage to the thermoregulatory center. During mechanical ventilation, SPONT mode was intermittently given to exercise the ability of spontaneous breathing, but the patient’s breathing was rapid after weaning, and the hope of weaning was small. This patient with poor outcomes may require long-term intensive care unit hospitalization.

## 4. Conclusion

Third nerve paralysis after embolization of ruptured anterior communicating aneurysm is very rare. To our knowledge, this is the first case of a patient in a coma with persistent pupil dilation after interventional embolization of ruptured anterior communicating aneurysm. It is difficult to determine the exact pathophysiological mechanism leading to this case, which brings great challenges to nursing work. This case report summarizes our nursing experience and related literature discussion, which may be helpful for the nursing of such patients. However, some interventions may not be universal due to the limitations of current care interventions because of individual differences. Therefore, more research on the treatment and care of patients with ONP caused by rupture of ACoA aneurysm is needed to support the results of this case study.

## Author contributions

**Writing – original draft:** Yong Cai.

**Writing – review & editing:** Xingming Zhong.
